# Impaired RNA Binding Does Not Prevent Histone Modification Changes in a FUS ALS/FTD Yeast Model

**DOI:** 10.17912/micropub.biology.000895

**Published:** 2023-09-08

**Authors:** Seth A. Bennett, Samantha N. Cobos, Elizaveta Son, Rianna Segal, Shana Mathew, Huda Yousuf, Mariana P. Torrente

**Affiliations:** 1 PhD. Program in Biochemistry, City University of New York - The Graduate Center, New York, NY, USA 10016; 2 PhD. Program in Chemistry, City University of New York - The Graduate Center, New York, NY, USA 10016; 3 Department of Chemistry and Biochemistry, Brooklyn College, Brooklyn, NY, USA 11210; 4 PhD. Programs in Chemistry, Biochemistry, and Biology, City University of New York - The Graduate Center, New York, NY, USA 10016

## Abstract

Mutations in the RNA-binding protein FUS are linked to amyotrophic lateral sclerosis and frontotemporal dementia (ALS/FTD). FUS mutants mislocalize and aggregate in dying neurons. We previously established that FUS proteinopathy is linked to changes in the histone modification landscape in a yeast ALS/FTD model. Here, we examine whether FUS’ RNA binding is necessary for this connection. We find that overexpression of a FUS mutant unable to bind RNA is still associated with reduced levels of H3S10ph, H3K14ac and H3K56ac. Hence, FUS’ ability to bind RNA is not required in the mechanism connecting FUS proteinopathy to altered histone post-translational modifications.

**Figure 1. RNA binding by FUS is not necessary for histone PTM dysregulation:**
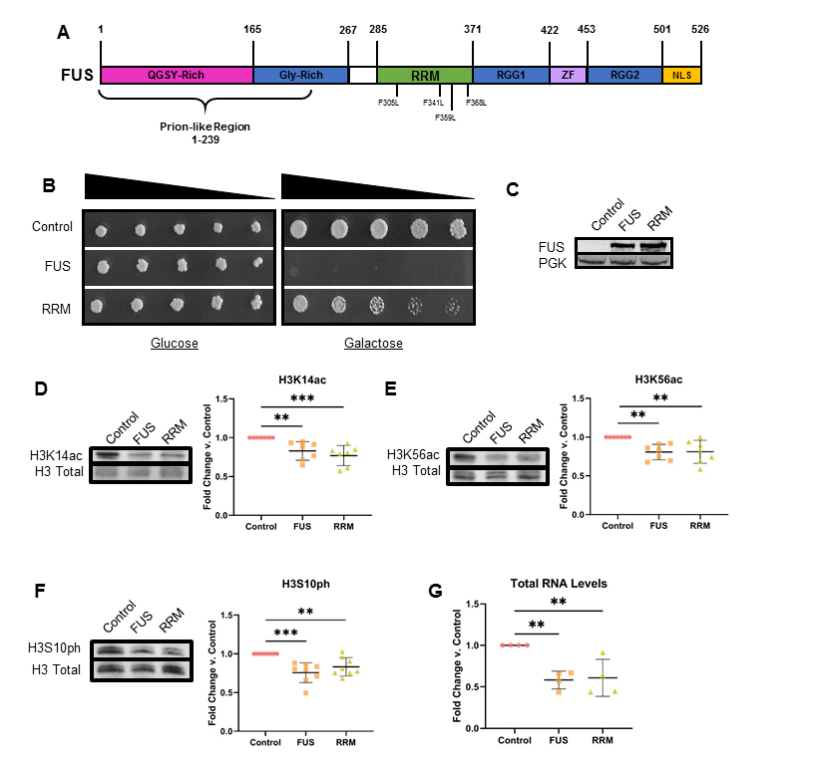
A) FUS domain architecture. Mutated phenylalanine sites in the FUS RRM domain used in this study are indicated. B) Serial dilution growth assays depict cell viability in glucose (non-inducing) and galactose (inducing) supplemented media (n =4). C) Western blots confirm expression of FUS and the RRM mutant (n = 4). Representative immunoblots showing the levels of D) H3K14ac (n = 7), E) H3K56ac (n = 7) and F) H3S10ph (n = 8) in yeast overexpressing FUS, RRM or a control plasmid. Column scatter graphs compiling multiple biological replicates are presented alongside blots. All graphs display the mean fold change in modification levels for each group based on densitometric analysis of Western blots. Total H3 was used as a loading control. G) Total RNA levels in control, FUS and RRM yeast. Graph displays the mean fold change in total RNA levels for each group (n = 4). Error bars indicate ±SD. *** = *p* < 0.001, ** = *p* < 0.01, * = *p <* 0.05.

## Description

**Introduction**

Amyotrophic lateral sclerosis (ALS) and frontotemporal dementia (FTD) form a ruinous neurodegenerative disease spectrum (Cobos et al. 2019) . On one end on this spectrum, ALS is characterized by the loss of upper and lower motor neurons, while on the other FTD is characterized by the degeneration of the frontal and temporal lobes of the brain (Ferrari et al. 2011) . With case numbers rising each year, there is an imperative need for a cure (Arthur et al. 2016) . There are a wide range of genetic features associated with ALS/FTD. For instance, mutations in the *SOD1, c9orf72, FUS, and TDP-43* genes have all been implicated in ALS/FTD pathology (An et al. 2019, Suk et al. 2020, Smeyers et al. 2021, Berdynski et al. 2022) .

*FUS* ( *Fused in Sarcoma* ) encodes for the RNA-binding protein FUS (Deng et al. 2014) . Typically a nuclear protein, FUS is involved in numerous cellular processes including RNA maturation, RNA binding, and DNA repair (Deng et al. 2014, Yamaguchi et al. 2016) . FUS is comprised of a QGSY-rich prion-like domain, a glycine-rich domain, two RGG-rich domains, an RNA recognition motif (RRM), a zinc finger, and a nuclear localization signal (Kang et al. 2019) . Mutations in FUS’ nuclear localization signal are pathogenic (Kwiatkowski et al. 2009, Vance et al. 2009, Bosco et al. 2010, Corrado et al. 2010, Van Langenhove et al. 2010, Deng et al. 2014, Hou et al. 2016) . These mutants lead to the formation of toxic cytoplasmic mutant FUS aggregates in decaying neurons (Bosco et al. 2010, Woulfe et al. 2010, An et al. 2019) . However, the exact mechanisms linking FUS aggregation to disease processes remain incompletely characterized.

Epigenetics refers to heritable changes to a phenotype occurring without directly affecting an organisms’ genetic background, typically through changes in chromatin structure and composition (Allis et al. 2016) . The basic structure of chromatin -termed nucleosome- consists of DNA wound around a histone octamer core (made out of two Histone H2A/H2B dimers and one Histone H3/H4 tetramer) (Cosgrove et al. 2004) . The N-terminal tails of the histones protrude out of the nucleosome, Heterochromatin is tightly wound and transcriptionally silent, while euchromatin is more open and transcriptionally active (Gibney et al. 2010) . Two notable forms of epigenetic regulation include the methylation of DNA and the post-translational modification (PTM) of histone proteins (Cosgrove et al. 2004, Allis et al. 2016) . Histone PTMs refer to the dynamic addition and removal of various chemical moieties to the N-terminal tails of histone proteins, which can either tighten or loosen the shape of chromatin, thereby affecting gene expression (Cosgrove et al. 2004) . Some of these moieties include methyl-, acetyl-, and phosphate groups that can be added by “writer” enzymes and removed by “eraser” enzymes (Strahl et al. 2000) . The presence or absence of certain modifications on histone tails makes a “code” that can then be read by “reader” enzymes which then trigger any number of cellular processes (Cosgrove et al. 2004) .

Previous work has revealed links between ALS/FTD and several histone modifying enzymes. For instance, the histone deacetylase (HDAC) HDAC1 mislocalizes to the cytoplasm in a FUS-ALS model (de Ruijter et al. 2003) . Moreover, RNAi silencing of FUS reduced the expression of HDAC6 mRNA (Kim et al. 2010) . Our own work has shown that levels of specific histone PTMs are significantly depleted in a *Saccharomyces cerevisiae* FUS ALS/FTD model (Chen et al. 2018) . Specifically, levels of H3S10ph, H3K14ac, and H3K56ac are reduced when compared to controls (Chen et al. 2018) . In addition, treatment with the pan-HDAC inhibitor Trichostatin A restored the levels of H3K14ac and H3K56ac while bypassing the toxic effects of FUS aggregation (Bennett et al. 2021) . Here, we explore whether FUS’ ability to bind RNA is necessary to elicit dysregulation of the epigenome. Surprisingly, we find that a FUS mutant bearing four point mutations ablating its ability to bind RNA (F305L, F341L, F359L and F368L) is still connected to histone PTM dysregulation in a yeast model. Hence, our results suggest that FUS’ association to alterations in the histone PTM landscape not dependent on RNA binding by FUS.

Results and Discussion

RNA binding by FUS is not necessary for histone PTM dysregulation

We have previously shown that the levels of H3S10ph, H3K14ac and H3K56ac are significantly decreased in a FUS overexpression ALS/FTD yeast model (Chen et al. 2018) . We wondered if RNA binding by FUS contributed to the mechanism resulting in dysregulation of histone PTMs. Individual domains in FUS’ sequence are responsible for a number of discrete functions; the prion-like domain is required for phase separation of FUS, while phosphorylation of specific sites inhibits phase separation (Owen et al. 2020) . Mutating conserved phenylalanine residues to leucine (F305L, F341L, F359L, F368L) in the RRM domain abolishes FUS’ ability to bind RNA ( **Figure 1A** ) (Sun et al. 2011) . Mutant FUS RRM (henceforth referred to as RRM) still aggregates (Sun et al. 2011) . but is markedly less toxic (Sun et al. 2011) . We transformed yeast with plasmids bearing FUS, RRM or a vector control under a galactose-inducible promoter. As previously reported (Sun et al. 2011) , we find wild type FUS overexpression results in a very marked growth suppression phenotype, but growth suppression is much less pronounced in yeast overexpressing RRM ( **Figure 1B** ). We confirmed that FUS expression was comparable for both constructs via western blotting ( **Figure 1C** ).

We assessed the levels of H3S10ph, H3K14ac and H3K56ac in yeast overexpressing FUS, RRM and a control plasmid. Surprisingly, histone acetylation levels on H3K14 and H3K56 were decreased in both FUS and RRM yeast compared to control ( **Figure 1D,E** ). We did not observe a statistically significant difference in the levels of these two PTMs between FUS and RRM yeast. Similarly, we observed a reduction in H3S10ph levels in both FUS and RRM yeast compared to the control, with no significant difference between FUS and RRM yeast ( **Figure 1F** ). Linear range assays for each of these antibodies is provided as Extended Data. These results suggest that dysregulation of histone PTMs occurs independently of FUS’ ability to bind RNA.

H3K56ac and H3K14ac are marks associated with the cell cycle and chromatin remodeling, while H3S10ph is a marker of mitosis (Topal et al. 2019; Duan and Smerdon 2014; Komar and Juszczynski 2020) . As such, we wondered whether FUS overexpression led to cell cycle arrest. Light microscopy revealed no statistically significant differences between budding versus non-budding cells in control, FUS, and RRM yeast (Extended Data), suggesting there are no widespread changes in cell cycle distribution among the strains.

Collectively, the dysregulated PTMs are involved in active gene expression (Chen et al. 2018) . Thus, we speculated that cells overexpressing FUS or RRM would exhibit changes in gene expression. To investigate this hypothesis, we chose a simple approach in which we quantitated total RNA levels in yeast overexpressing FUS or RRM and compared them to yeast overexpressing a control plasmid. While RNA levels are not an ideal proxy for gene expression, they offer a very rough measure of transcriptional activity. In agreement with our histone PTM findings, we observe a roughly 50% decrease in total RNA in both FUS and RRM yeast, with no significant difference between FUS and RRM yeast ( **Figure 1G** ). Notably, we observed this effect despite protein overexpression. These results further support that FUS’ connection to PTM dysregulation and subsequent impact on total cellular RNA levels is independent of FUS-RNA binding.

Altogether, we find that histone PTM dysregulation is independent of RNA binding by FUS in a yeast overexpression model. It is important to note that some of the histone modifications we study are cell cycle dependent, and thus, without flow cytometry experiments we cannot completely discard the possibility that overexpression of FUS or RRM is altering cell cycle distribution. Moreover, our studies are limited to yeast models, and thus, mechanistic verification in other model systems is still necessary (Jiang et al. 2006). Proteomic analysis of putative FUS binding partners in SH-SY5Y cells revealed a significant number of RNA processing, splicing and binding proteins (Kamelgarn et al. 2016) . Hence, FUS aggregation could be impacting RNA processes independently of its own RNA binding abilities. Furthermore, other FUS domains and functions could be connecting FUS aggregation to the epigenome. For instance, FUS also binds histone H4 and H2A and H2B variants in SH-SY5Y cells (Kamelgarn et al. 2016) . This further underscores the relationship between FUS aggregates and the epigenome.

**Conclusions**

Specific histone PTM alterations are connected to FUS proteinopathy in yeast models, though the molecular mechanisms behind this association are unclear. RNA-binding through the RRM domain is not necessary for FUS aggregate formation in yeast, but it is necessary to elicit a growth suppression phenotype. Surprisingly, we have shown that FUS does not need to bind RNA to elicit changes in histone PTM levels or global RNA levels. Hence, it appears that histone PTM changes are linked to aggregation of FUS. Overall, our results suggest aggregation – rather than defects in than RNA binding – as an important feature connecting FUS to the epigenome in ALS/FTD.

## Methods

**Materials and Methods**

Yeast Strains, Media and Plasmids All yeast were W303a ( *MATa, can1-100,his3-11, 15,leu2,3,11,12,trp1-1,ura3-1,ade2-1).* (Sanchez et al. 1990) Yeast were grown in synthetic dropout medium (Clonetech Laboratories, Mountain View, CA) supplemented with 2% glucose, raffinose or galactose. The plasmids 426Gal-FUS-RRM-YFP (Addgene plasmid no. 29608), 426Gal-FUS-YFP (Addgene plasmid no. 20592) and paG426Gal-ccdB (Addgene plasmid no. 14155) were gifts from A. Gitler and Susan Lindquist, respectively. Yeast were transformed using standard poly(ethylene glycol) and lithium acetate protocols (Bennett et al. 2019) .

Protein Overexpression. Yeast strains were grown to saturation overnight in raffinose-supplemented dropout media at 30 °C and 200 rpm. Overnight cultures were then diluted to an OD _600_ of 0.30 in galactose-supplemented synthetic dropout media and induced for 5 hours at 30°C. Yeast cultures were then standardized to the lowest OD _600_ . Cells were then pelleted at 850 rcf at 4°C and washed three times with sterile distilled water and harvested. The supernatant was removed and the pellets were flash frozen in liquid nitrogen and stored at -80°C.

Serial dilution growth assays. Yeast were grown to saturation overnight in raffinose-supplemented dropout media at 30°C. Overnight cultures were diluted 2-fold, then serially diluted 5-fold. Yeast were spotted onto solid synthetic dropout medium containing glucose or galactose with a pin-frogger. Yeast were grown at 30°C for 3 to 4 days before imaging.

Western blotting. Western blotting was performed as previously described (Bennett et al. 2019) . Briefly, frozen yeast cell pellets were thawed and treated with 0.2 M NaOH for 10 minutes on ice, pelleted again, and subsequently resuspended in 100 mL of 1X SDS sample buffer and boiled for 10 minutes. Cell lysates were separated using SDS-PAGE and then transferred to a PVDF membrane (EMD Millipore). Membranes were blocked using LI-COR blocking buffer (LI-COR Biosciences, Lincoln, NE) for 1 hour at RT. Membranes were incubated with primary antibodies at 4 °C overnight. Primary antibodies used were: rabbit anti-FUS polyclonal (Bethyl Laboratories, Montgomery, TX; cat. no. A300-302A, 1:1,000 dilution), mouse anti-PGK monoclonal (Novex, Frederick, MD; cat. no. 459250, 1:2,000 dilution), mouse anti-H3 total (Abcam, Cambridge, MA; cat. no. ab24834, 1:2,000 dilution), rabbit anti-H3S10ph (Abcam, Cambridge, MA; cat. no. ab5176, 1:1,000 dilution), rabbit anti-H3K14ac (Millipore, cat. no. 07-353, 1:2,000 dilution), and rabbit anti-H3K56ac (Active Motif, Carlsbad, CA; cat. no. 39281, 1:5,000 dilution). Blots were processed using goat anti-mouse and anti-rabbit secondary antibodies from LI-COR Biosciences (both at 1:20,000 dilution) and imaged using an Odyssey Fc imaging system (LI-COR Biosciences). All immunoblotting experiments were independently repeated a minimum of three times. Densitometric analysis of Western blots was performed using Image Studio (LI-COR Biosciences). The signals obtained for histone modifications were normalized to their respective total H3 signals (modification/total H3). These values were then compared with untreated control-sample values to obtain fold change values (sample/control), which were used for statistical analysis.

Microscopy. Control, FUS, and RRM yeast pellets were thawed and resuspended in 70% (w/v) ethanol for 30 minutes at room temperature. Yeast were pelleted again and washed twice with PBS. Pellets were then resuspended in a small volume of PBS and imaged immediately. Slides were observed using a wide-field Axio Observer 7 Inverted Microscope (Zeiss) with a x63/1.4-numerical aperture (NA) Plan-Apochromat (oil immersion) objective. The resulting images were processed using both Zen Microscopy Software (Zen) and ImageJ (Fiji). Average percentages of budding vs. non-budding cells were calculated from five representative images for three biological replicates of each strain.

RNA Purification. Frozen pellets were thawed and then treated with zymolyase 20-T (USBiologicals, Salem, Massachusetts; cat. No. Z1000-250MG). Total RNA was then purified from samples using a Qiagen RNeasy Miniprep Kit (Qiagen, Hilden, Germany; cat. No. 74104). RNA concentrations were measured using a NanoDrop Lite (ThermoFisher Scientific, Waltham, Massachusetts). RNA concentrations from FUS and RRM yeast were then compared to control yeast to obtain fold change values (sample/control), which were used for statistical analysis.

Statistical Analysis. Statistical analysis of data was performed in GraphPad Prism 9 using the built-in stats package (GraphPad Software Inc., California). Significant differences between nuclear intensity and histone modifying enzymes levels were determined using Welch’s *t* test with *p* = 0.05 as the cutoff. Significant differences between FUS, RRM, and control groups were determined using one-way ANOVA followed by Tukey’s test for pairwise comparison of the group means with *p* = 0.05 as the cutoff for significance. Error bars on the graphs represent standard deviation (SD) calculated from values obtained in the data analysis steps described above. All graphs were constructed with GraphPad Prism 9 (GraphPad Software Inc., California) (Swift 1997) .

## Extended Data

Description: Linear Range Assays for Selected Antibodies and Light Microscopy. Resource Type: Dataset. DOI: 10.22002/s6mgq-cqe22

## References

[R1] Allis CD, Jenuwein T (2016). The molecular hallmarks of epigenetic control.. Nat Rev Genet.

[R2] An H, Skelt L, Notaro A, Highley JR, Fox AH, La Bella V, Buchman VL, Shelkovnikova TA (2019). ALS-linked FUS mutations confer loss and gain of function in the nucleus by promoting excessive formation of dysfunctional paraspeckles.. Acta Neuropathol Commun.

[R3] Arthur KC, Calvo A, Price TR, Geiger JT, Chiò A, Traynor BJ (2016). Projected increase in amyotrophic lateral sclerosis from 2015 to 2040.. Nat Commun.

[R4] Bennett SA, Cobos SN, Meykler M, Fallah M, Rana N, Chen K, Torrente MP (2019). Characterizing Histone Post-translational Modification Alterations in Yeast Neurodegenerative Proteinopathy Models.. J Vis Exp.

[R5] Bennett SA, Cobos SN, Mirzakandova M, Fallah M, Son E, Angelakakis G, Rana N, Hugais M, Torrente MP (2021). Trichostatin A Relieves Growth Suppression and Restores Histone Acetylation at Specific Sites in a FUS ALS/FTD Yeast Model.. Biochemistry.

[R6] Berdyński M, Miszta P, Safranow K, Andersen PM, Morita M, Filipek S, Żekanowski C, Kuźma-Kozakiewicz M (2022). SOD1 mutations associated with amyotrophic lateral sclerosis analysis of variant severity.. Sci Rep.

[R7] Bosco DA, Lemay N, Ko HK, Zhou H, Burke C, Kwiatkowski TJ Jr, Sapp P, McKenna-Yasek D, Brown RH Jr, Hayward LJ (2010). Mutant FUS proteins that cause amyotrophic lateral sclerosis incorporate into stress granules.. Hum Mol Genet.

[R8] Chen K, Bennett SA, Rana N, Yousuf H, Said M, Taaseen S, Mendo N, Meltser SM, Torrente MP (2018). Neurodegenerative Disease Proteinopathies Are Connected to Distinct Histone Post-translational Modification Landscapes.. ACS Chem Neurosci.

[R9] Cobos SN, Bennett SA, Torrente MP (2018). The impact of histone post-translational modifications in neurodegenerative diseases.. Biochim Biophys Acta Mol Basis Dis.

[R10] Corrado L, Del Bo R, Castellotti B, Ratti A, Cereda C, Penco S, Sorarù G, Carlomagno Y, Ghezzi S, Pensato V, Colombrita C, Gagliardi S, Cozzi L, Orsetti V, Mancuso M, Siciliano G, Mazzini L, Comi GP, Gellera C, Ceroni M, D'Alfonso S, Silani V (2009). Mutations of FUS gene in sporadic amyotrophic lateral sclerosis.. J Med Genet.

[R11] Cosgrove MS, Boeke JD, Wolberger C (2004). Regulated nucleosome mobility and the histone code.. Nat Struct Mol Biol.

[R12] de Ruijter AJ, van Gennip AH, Caron HN, Kemp S, van Kuilenburg AB (2003). Histone deacetylases (HDACs): characterization of the classical HDAC family.. Biochem J.

[R13] Deng H, Gao K, Jankovic J (2014). The role of FUS gene variants in neurodegenerative diseases.. Nat Rev Neurol.

[R14] Duan MR, Smerdon MJ (2014). Histone H3 lysine 14 (H3K14) acetylation facilitates DNA repair in a positioned nucleosome by stabilizing the binding of the chromatin Remodeler RSC (Remodels Structure of Chromatin).. J Biol Chem.

[R15] Ferrari R, Kapogiannis D, Huey ED, Momeni P (2011). FTD and ALS: a tale of two diseases.. Curr Alzheimer Res.

[R16] Gibney ER, Nolan CM (2010). Epigenetics and gene expression.. Heredity (Edinb).

[R17] Hou L, Jiao B, Xiao T, Zhou L, Zhou Z, Du J, Yan X, Wang J, Tang B, Shen L (2016). Screening of SOD1, FUS and TARDBP genes in patients with amyotrophic lateral sclerosis in central-southern China.. Sci Rep.

[R18] Kamelgarn M, Chen J, Kuang L, Arenas A, Zhai J, Zhu H, Gal J (2016). Proteomic analysis of FUS interacting proteins provides insights into FUS function and its role in ALS.. Biochim Biophys Acta.

[R19] Kang J, Lim L, Song J (2019). ATP binds and inhibits the neurodegeneration-associated fibrillization of the FUS RRM domain.. Commun Biol.

[R20] Kim SH, Shanware NP, Bowler MJ, Tibbetts RS (2010). Amyotrophic lateral sclerosis-associated proteins TDP-43 and FUS/TLS function in a common biochemical complex to co-regulate HDAC6 mRNA.. J Biol Chem.

[R21] Komar D, Juszczynski P (2020). Rebelled epigenome: histone H3S10 phosphorylation and H3S10 kinases in cancer biology and therapy.. Clin Epigenetics.

[R22] Kwiatkowski TJ Jr, Bosco DA, Leclerc AL, Tamrazian E, Vanderburg CR, Russ C, Davis A, Gilchrist J, Kasarskis EJ, Munsat T, Valdmanis P, Rouleau GA, Hosler BA, Cortelli P, de Jong PJ, Yoshinaga Y, Haines JL, Pericak-Vance MA, Yan J, Ticozzi N, Siddique T, McKenna-Yasek D, Sapp PC, Horvitz HR, Landers JE, Brown RH Jr (2009). Mutations in the FUS/TLS gene on chromosome 16 cause familial amyotrophic lateral sclerosis.. Science.

[R23] Owen I, Rhoads S, Yee D, Wyne H, Gery K, Hannula I, Sundrum M, Shewmaker F (2020). The prion-like domain of Fused in Sarcoma is phosphorylated by multiple kinases affecting liquid- and solid-phase transitions.. Mol Biol Cell.

[R24] Sanchez Y, Lindquist SL (1990). HSP104 required for induced thermotolerance.. Science.

[R25] Smeyers J, Banchi EG, Latouche M (2021). C9ORF72: What It Is, What It Does, and Why It Matters.. Front Cell Neurosci.

[R26] Strahl BD, Allis CD (2000). The language of covalent histone modifications.. Nature.

[R27] Suk TR, Rousseaux MWC (2020). The role of TDP-43 mislocalization in amyotrophic lateral sclerosis.. Mol Neurodegener.

[R28] Sun Z, Diaz Z, Fang X, Hart MP, Chesi A, Shorter J, Gitler AD (2011). Molecular determinants and genetic modifiers of aggregation and toxicity for the ALS disease protein FUS/TLS.. PLoS Biol.

[R29] Swift Mary L. (1997). GraphPad Prism, Data Analysis, and Scientific Graphing. Journal of Chemical Information and Computer Sciences.

[R30] Topal S, Vasseur P, Radman-Livaja M, Peterson CL (2019). Distinct transcriptional roles for Histone H3-K56 acetylation during the cell cycle in Yeast.. Nat Commun.

[R31] Van Langenhove T, van der Zee J, Sleegers K, Engelborghs S, Vandenberghe R, Gijselinck I, Van den Broeck M, Mattheijssens M, Peeters K, De Deyn PP, Cruts M, Van Broeckhoven C (2010). Genetic contribution of FUS to frontotemporal lobar degeneration.. Neurology.

[R32] Vance C, Rogelj B, Hortobágyi T, De Vos KJ, Nishimura AL, Sreedharan J, Hu X, Smith B, Ruddy D, Wright P, Ganesalingam J, Williams KL, Tripathi V, Al-Saraj S, Al-Chalabi A, Leigh PN, Blair IP, Nicholson G, de Belleroche J, Gallo JM, Miller CC, Shaw CE (2009). Mutations in FUS, an RNA processing protein, cause familial amyotrophic lateral sclerosis type 6.. Science.

[R33] Woulfe J, Gray DA, Mackenzie IR (2009). FUS-immunoreactive intranuclear inclusions in neurodegenerative disease.. Brain Pathol.

[R34] Yamaguchi A, Takanashi K (2016). FUS interacts with nuclear matrix-associated protein SAFB1 as well as Matrin3 to regulate splicing and ligand-mediated transcription.. Sci Rep.

